# Problem solving, impulse control and planning in patients with early- and late-stage Huntington’s disease

**DOI:** 10.1007/s00406-016-0707-4

**Published:** 2016-07-02

**Authors:** Sabrina Mörkl, Nicole J. Müller, Claudia Blesl, Leonora Wilkinson, Adelina Tmava, Walter Wurm, Anna K. Holl, Annamaria Painold

**Affiliations:** 1 Department of Psychiatry, Medical University of Graz, Auenbruggerplatz 31/1, 8036 Graz, Austria; 2 Behavioral Neurology Unit, National Institute of Neurological Disorders and Stroke, National Institutes of Health, 10 Center Dr., MSC 1440, Bethesda, MD 20892-1440 USA

**Keywords:** Huntington’s disease, Executive function, Tower of London, Neuropsychology, Disease severity

## Abstract

Sub-domains of executive functions, including problems with planning, accuracy, impulsivity, and inhibition, are core features of Huntington’s disease. It is known that the decline of cognitive function in Huntington’s disease is related to the anatomical progression of pathology in the basal ganglia. However, it remains to be determined whether the severity of executive dysfunction depends on the stage of the disease. To examine the severity of sub-domains of executive dysfunction in early- and late-stage Huntington’s disease, we studied performance in the Tower of London task of two groups of Huntington’s disease patients (Group 1: early, *n* = 23, and Group 2: late stage*, n* = 29), as well as a third group of age, education, and IQ matched healthy controls (*n* = 34). During the task, we measured the total number of problems solved, total planning time, and total number of breaks taken. One aspect of executive function indexed by the number of solved problems seems to progress in the course of the disease. Late-stage Huntington’s disease patients scored significantly worse than early-stage patients and controls, and early-stage patients scored significantly worse than controls on this measure of accuracy. In contrast, late- and early-stage HD patients did not differ in terms of planning time and number of breaks. Early- and late-stage HD pathology has a different impact on executive sub-domains. While accuracy differs between early- and late-stage HD patients, other domains like planning time and number of breaks do not. Striatal degeneration, which is a characteristic feature of the disease, might not affect all aspects of executive function in HD.

## Introduction

Neuropsychological deficits caused by neurodegenerative conditions, such as dementia, multiple sclerosis, amyotrophic lateral sclerosis and Huntington’s disease (HD), significantly impair patients’ abilities and affect their quality of life [1–4].

HD is an autosomal-dominant trinucleotide repeat disorder with early signs of cognitive deterioration. The underlying defect in HD is an elongated gene, which produces a protein called huntingtin, leading to neural apoptosis especially in the striatum. In patients with HD, cognitive dysfunction may precede the first neurologic signs by up to 10 years [5–7]. The first signs of cognitive dysfunction are usually the deterioration of executive functions and psychomotor speed, which are prominent signs in the preclinical stages and in early disease stages. This ‘frontal’ pattern of cognitive deterioration in HD is considered to result from early neuronal death in the caudate, which leads to dysfunction in fronto-striatal circuits [8]. Several MRI studies showed consistent degeneration of the striatum (caudate nucleus and putamen) already in presymptomatic patients [9–11] and in symptomatic stages of the disease [5, 12].

Due to this progressive degeneration in specific parts of the brain, it has been assumed that clinical features would decrease linearly too. While several studies on the impairment of cognitive functions in prodromal and early stages of the disease have been conducted [5–7, 13–15], cognitive function in late stages has been assessed rarely. The presence of increasing neuronal cell loss especially in the striatum is expected to lead to global dementia, and it follows that late-stage patients should therefore show a pathologic performance on all cognitive functions.

However, this assumption was called in question by studies that reported a nonlinear degeneration of some cognitive functions. Beste et al. [16] reported in their study on the assessment of auditory sensory memory that symptomatic HD patients performed better than presymptomatic HD patients and healthy controls on an auditory signal detection task. They proposed that the better performance of symptomatic patients occurred due to an increased activity of the NMDA receptor system, which occurs regularly in patients experiencing neuronal cell death. Papoutsi et al. [15] highlighted that it is unknown if the development of cognitive decline is comparable with that of topographical changes of the striatum. They also suggested that cognitive decline may be compensated at a certain point and that brain changes and cognitive changes may not develop simultaneously. Due to the small number of studies on cognitive function in severe stages of HD, it remains unclear whether all aspects of cognitive function are worse in late-stage HD patients compared to early-stage HD patients.

The Tower of London (ToL) [17] is a neuropsychological test, sensitive to executive functions like mental problem solving, planning, behavioural inhibition, and impulse control. The executive function problem solving (accuracy) is measured by the total number of solved problems in four levels of difficulty. Behavioural inhibition or impulse control is reflected by the measure of planning time (in other studies also called reaction time or latency), which is the time from the presentation of the start board to the first movement. The number of breaks (pausing during the process of problem solving) is an indicator for problem reflection and planning. Particularly, patients with affections of the frontal lobe tend to rash and impulsive decisions. This behaviour is a sign of increased impulsivity and decreased ability of self-control [17].

**Fig. 1:**
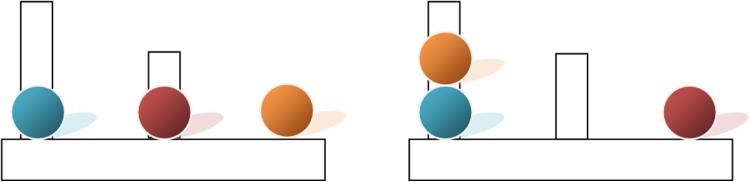
Illustration of the ToL task—example for a two-move problem

Participants are given a start and a goal board containing three pegs of descending lengths and three balls; they are instructed to transform the start board ball arrangement to the goal board ball arrangement using the smallest number of single-ball movements possible and observing various limitations (Fig. 1).

The ToL has been used in one study of early-stage HD patients [18] and in one study of both presymptomatic and early patients [19]. Watkins and colleagues reported significantly worse performance of early-stage HD patients relative to controls for the problem solving during the so-called ‘one-touch’-ToL, which is a modified version of the ToL, that minimizes motor demands of the task [18]. In an fMRI adapted version of the task, Unschuld et al. [19] reported a significantly worse accuracy in early-stage HD patients relative to controls but no significant differences between the three groups for planning time. As both of the above studies of ToL performance in HD were conducted with early-stage HD patients, their findings do not inform us how late-stage HD patients would score on the task. If the topographical changes of the striatum associated with the late stage of the disease are directly related to the decline of executive function, it follows that accuracy and reaction time should be severely impaired, in late-stage HD patients relative to controls and early-stage patients. In contrast, if accuracy and planning time would not differ between early- and late-stage HD patients, then it would indicate that other neurophysiological substrates might contribute to balance executive dysfunctions due to neuronal cell death in the caudate in HD. With respect to the ToL, there are two potential outcomes for late-stage HD patients: *I)* presuming that cognitive functions deteriorate linearly and that cognition worsens according to the topographical changes in the striatum; we would expect a significantly worse result for accuracy and a much longer reaction time in late-stage HD patients relative to controls and early-stage patients as well combined with a significant but less robust impairment of performance in early-stage patients relative to controls. *II)* If we presume that the development of cognitive decline is *not* comparable with that of topographical changes of the striatum, we may find any other results in severe HD patients as well as in those with early stage.

The aim of the present study was to examine sub-aspects of executive functioning in early-stage and late-stage HD. We assessed both groups of HD patients using the ToL task; this included an assessment of number of solved problems, planning time and number of breaks. In early-stage HD it is established that in preclinical stage, HD gene mutation carriers are nearly free of symptoms, regardless of the existence of neurodegeneration. Functional brain imaging studies suggest a neural compensation in early-stage HD, where the brain, in response to neurodegeneration, develops mechanisms of functional reorganization in order to maintain cognitive performance [15, 20]. We hypothesized that executive functions would partially differ between the early- and late-stage HD group.

## Methods

### Participants

Fifty-two patients (32 male) with genetically proven HD aged between 25 and 69 (*M* = 47.69, *SD* = 11.20) were studied. The patients were recruited from the HD clinic at our Department of Psychiatry. Patients were in stage one to stage four of the disease, rated with Shoulson’s clinical stages [21]. To compare early affected patients with those in later stages, we divided them into two groups based on disease severity: early-stage group (stage 1 and 2, *n* = 23, male = 15) and late-stage group (stage 3 and 4, *n* = 29, male = 17). As stage division according to the clinical stages of Shoulson [21] depends on a number of clinical characteristics, disease duration alone is not used to divide the patients in an early- and late-stage group. Therefore, individual early- and late-stage HD patients could have a similar duration of illness.

The Unified Huntington’s Disease Motor Score Rating Scale (UHDRS motor) [22] was employed for the assessment of motor symptoms. The UHDRS cognitive score was calculated as the sum of the scores of the verbal fluency test, the symbol digit modalities test, and the Stroop test (Stroop colour, words, and inhibition) [22]. The total functional capacity (TFC) and the mini-mental state examination (MMSE) were assessed in all patients. Disease duration ranged from 1 to 7 years (*M* = 2.48, SD = 2.00) in the early-stage group and from 1 to 17 years (*M* = 6.93, SD = 4.33) in the late-stage group. All patients presented with a positive genetic test for the HD gene with a CAG repeat expansion between 40 and 58. For all patients, an estimate of premorbid IQ was obtained with the MWT-B (Mehrfachwahl-Wortschatz-Intelligenz test) [23].

At the time of testing, 32/52 patients were treated with disease-related medication. Two late-stage HD patients were treated with antipsychotics, mainly for treatment of choreatic movements and personality changes, 19 were treated with a combination of antipsychotics and antidepressants (one early-stage patient and 18 late-stage patients), and 11 were treated with antidepressants (nine early-stage HD patients and two late-stage HD patients). None of the patients showed any signs of depression at the time of assessment. Seven early-stage HD patients and 15 late-stage HD patients had previous major depression, five early-stage HD and five late-stage HD patients had previous minor depression.

Thirty-four healthy volunteers (19 male) aged between 18 and 77 (*M* = 49.06, *SD* = 17.38) took part in the study. Controls were recruited at the Campus of the Karl-Franzens-University and at the Medical University of Graz through word of mouth and flyers posted on the university campus. Prior to participation, they were screened for suitability. None of the medication-free controls had any neurological disorder, psychiatric illness, head injury, or history of alcohol or drug abuse. For all patients and controls, comorbid psychiatric diagnoses have been excluded in a diagnostic interview which was performed by our experienced consultant psychiatrist (AH).

Information about controls and HD patients is presented in Table 1. The study was approved by the local Ethics Committee of our Medical University. Informed consent was obtained prior to participation in the study from all participants.

**Table 1:** Demographic information for patients with early- and late-stage Huntington’s disease (HD) and controls and clinical characteristics of the patients

Group	Late-stage HD (*n* = 29)	Early-stage HD (*n* = 23)	Controls (*n* = 34)	*p*
	Mean (SD)	Mean (SD)	Mean (SD)	
Age (years)	49.21 (11.13)	45.78 (11.23)	49.06 (17.38)	.62
Education (years)	12.24 (1.73)	12.00 (2.32)	12.44 (2.76)	.78
Male/female participants	17/12	15/8	19/15	.88
Disease duration (years)	6.93 (4.33)	2.48 (2.00)		<.001
Genetic burden score	481.50 (106.47)	413.52 (90.41)		.03
CAG repeats	46.30 (4.87)	45.45 (3.74)		.52
Mini-mental state examination (0–30)	23.79 (4.38)	26.90 (3.43)		.02
Premorbid IQ	104.17 (13.83)	108.39 (11.13)		.33
Unified Huntington’s disease motor score rating scale (0–124)	38.76 (14.70)	20.04 (13.46)		<.001
Unified Huntington’s disease cognitive rating scale	128.54 (49.25)	210.22 (69.13)		<.001
Total functional capacity (0–13)	6.29 (2.72)	11.39 (2.39)		<.001

*SD* standard deviation, *NS* not significant

### Methods

We used the German version of the ToL [17]. In the standard version of this task [24], participants are given two boards (start board and goal board), each board containing three pegs of descending lengths and three balls: one red, one yellow, and one blue ball. Participants are instructed to transform the start board ball arrangement to look exactly like the goal board ball arrangement using the smallest number of single-ball movements possible. There is only one correct approach for each task. There are several limitations on the ball movements: it is not allowed to place more balls on a peg than it can hold; there cannot be more than one ball off a peg at the same time; and a ball cannot be moved if it is underneath another ball. Participants were told not to start until they had found the right solution.

Participants had to solve four sets of each five tasks of increasing difficulty (three-move problems, four-move problems, five-move problems, and six-move problems). For this study, we recorded if participants were able to arrange the balls with the smallest number of ball movements. A task was only considered as correct if the participant managed to use the smallest number of movements. Additionally, the planning time and the number of breaks were recorded.

## Results

### Participants

The groups of late-stage HD, early-stage HD and controls were matched for age [*F*_(2,83)_ = .48, *p* = .62], education [*F*_(2,83)_ = .25, *p* = .78], IQ [*F*_(2,83)_ = .99, *p* = .33], and sex [*χ*_(2)_^2^ = .50, *p* = .88].

### Number of solved problems

**Fig. 2:**
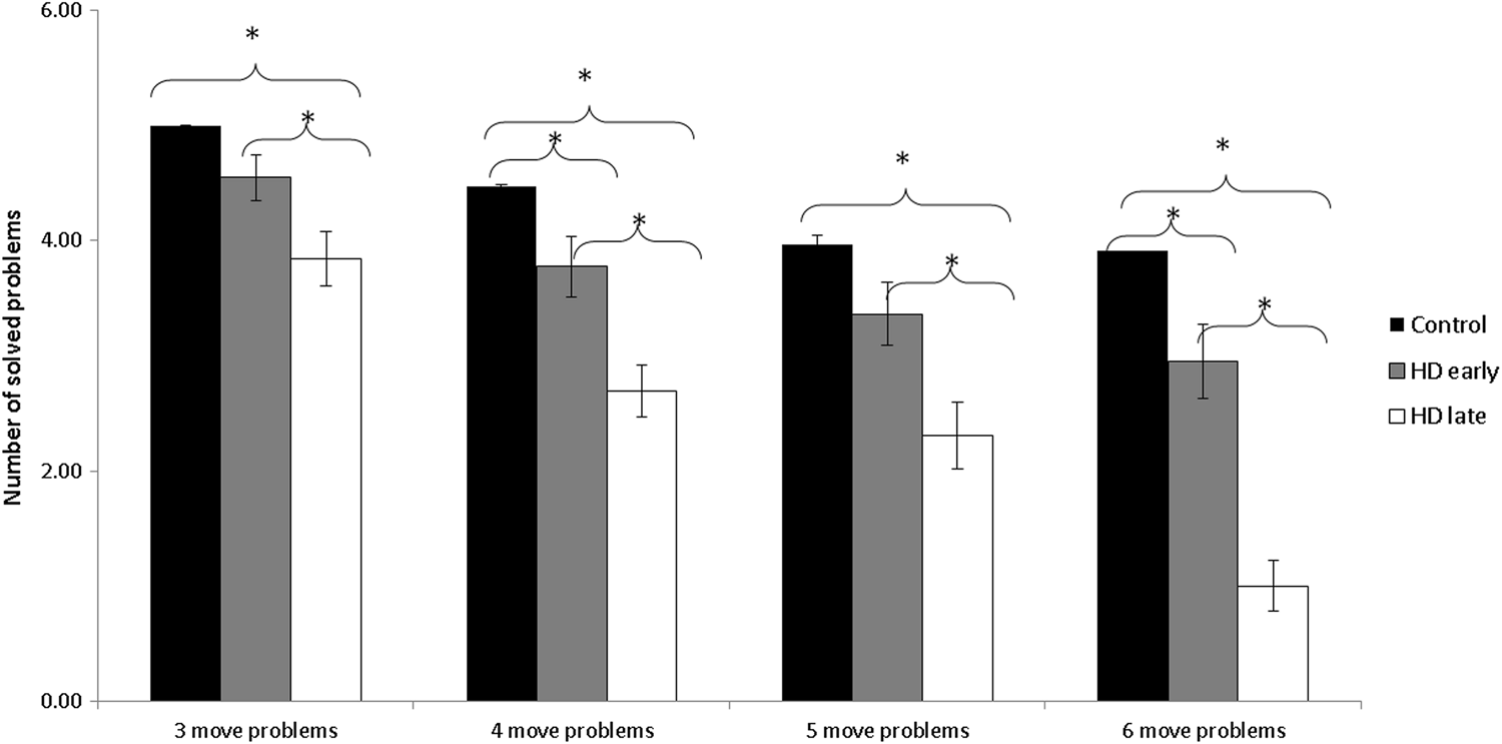
Number of solved problems for three-, four-, five- and six-move problems, plotted for late-stage HD patients, early-stage HD patients and controls. *Error bars* represent standard errors. *Asterisks* indicate where the comparison between the groups was significant. *p < .05

Figure 2 depicts the number of solved problems for three, four, five, and six-move problems of the ToL plotted separately for the three groups. To determine the effect of HD disease severity on accuracy during the ToL task, an ANOVA was performed on mean number of solved problems with level of Difficulty (three-move problem vs. four-move problems vs. Five-move problems vs. six-move problems) as a within-subjects variable and Group as a between-group variable (late-stage HD vs. early-stage HD vs. controls). This analysis revealed significantly main effects of Difficulty [*F*_(3,249)_ = 76.85, *p* < .001], Group [*F*_(2)_ = 24.93, *p* < .001], and a significant interaction between Group x Difficulty [*F*_(6,249)_ = 4.87, *p* < .001]. Therefore, the performance of all three groups across all four levels of difficulty was significantly different.

In light of the significant interaction between Group x Difficulty, we compared the mean number of solved problems between each of the three groups and at each of the four levels of difficulty in performing a post hoc test (Tukey). For the comparison between early- and late-stage HD patients, a significant difference was found in following conditions: three-move problems [Tukey; *p* = .04], four-move problems [Tukey; *p* = .02], five-move problems [Tukey; *p* = .03], and six-move problems [Tukey, *p* < .001]. For the comparison between controls and late-stage HD patients in all four conditions, late-stage HD patients scored significantly worse relative to controls (three-move problems [Tukey, *p* < .001]; four-move problems [Tukey, *p* < .001], five-move problems [Tukey, *p* < .001], and six-move problems [Tukey*, p* < .001]). In the comparison between early-stage HD patients and controls, early-stage HD patients scored significantly worse in following conditions: four-move problems [Tukey, *p* = .05] and six-move problems [Tukey, *p* = .03]).

### Planning time

**Fig. 3:**
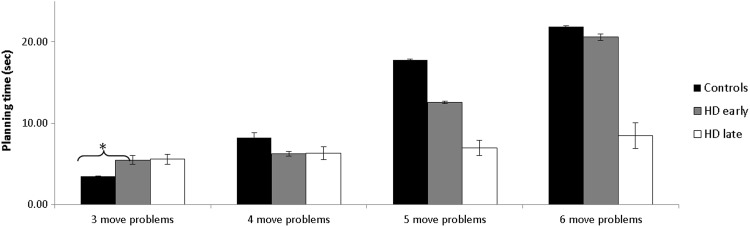
Total planning time for three-, four-, five- and six-move problems, plotted separately for late-stage HD patients, early-stage HD patients and controls. *Error bars* represent standard errors. *Asterisks* indicate where the comparison between the groups was significant. *p < .05

Figure 3 shows the total planning time for three-, four-, five-, and six-move problems of the ToL plotted separately for the three groups. To determine the effect of HD disease severity on planning time, an ANOVA was performed with Difficulty as a within-subjects variable and Group as a between-group variable. This analysis revealed significantly main effects of Difficulty [*F*_(3,249)_ = 28.81, *p* < .001], Group [*F*_(2)_ = 4.11, *p* = .02], and a significant interaction between Group × Difficulty [*F*_(6,249)_ = 5.61, *p* < .001].

In light of the interaction between Group × Difficulty, we compared the mean planning time between each of the three groups and at each of the four levels of difficulty. For the comparison between controls and early-stage HD patients, a significant difference was found in the planning time of three-move problems [Tukey, *p* = .02], where early-stage patients needed more time to start. For the comparison of planning time between controls and late-stage HD patients as well as in the comparison of both groups of HD patients for planning time, no significant differences were found.

### Number of breaks

**Fig. 4:**
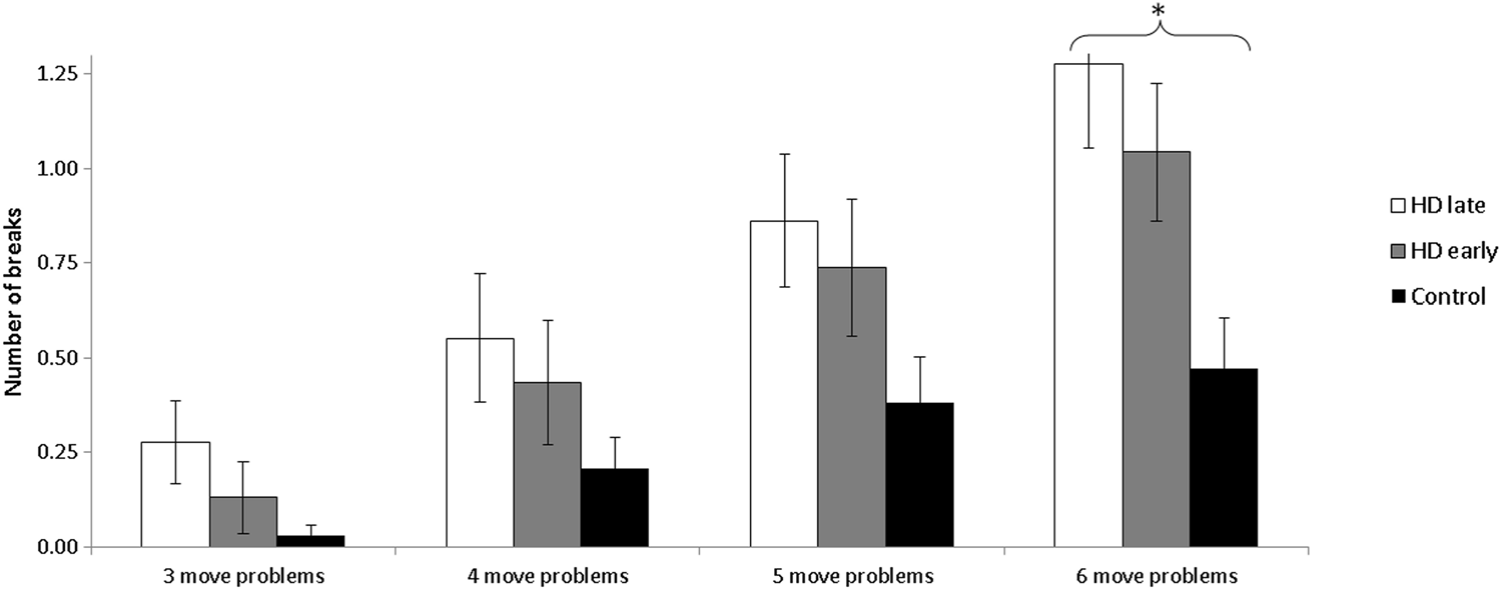
Number of breaks for three-, four-, five- and six-move problems, plotted separately for late-stage HD patients, early-stage HD patients and controls. *Error bars* represent standard errors. *Asterisks* indicate where the comparison between the groups was significant. *p < .05

Figure 4 depicts the total number of breaks for three-, four-, five- and six-move problems of the ToL plotted separately for the two patient groups and for the three groups. To determine the effect of HD disease severity on number of breaks during the ToL task, an ANOVA was performed on number of breaks with Difficulty as a within-subjects variable and Group as a between-groups variable. This analysis revealed significantly main effects of Difficulty [*F*_(3,249)_ = 27.36, *p* < .001] and Group [*F*_(2)_ = 5.57, *p* = .005]. Furthermore, we calculated the total number of breaks across all the four difficulty conditions.

For the comparison between controls and early-stage HD patients as well as for the comparison between late- and early-stage HD patients, there were no differences in the number of brakes in three-, four-, five- and six-move problems. For the comparison between controls and late-stage HD patients, late-stage HD patients differed significantly from controls [Tukey, *p* = .04].

### Correlations

To explore whether within the groups of early-stage and late-stage HD patients there was any relationship between task performance and disease parameters, correlations were calculated between total number of solved problems, total planning time, total number of breaks and the main clinical and demographic measures (age, years of education, disease duration, genetic burden score, CAG repeats, MMSE, premorbid IQ, UHDRS motor scale, UHDRS cognitive scores, TFC). Parametric correlation coefficients (Pearson’s *r*) were calculated; significance was assessed using a 2-tailed test. Table 2 shows the significant correlations of late-stage and early-stage HD patients. For the group of early-stage HD patients, the total number of solved problems was significantly positively correlated with MMSE, premorbid IQ, and UHDRS cognitive score and negatively correlated with disease duration and UHDRS motor score, indicating the less patients were handicapped from their disease the better they scored on the ToL. For the group of late-stage HD patients, the total number of solved problems was significantly positively correlated with the UHDRS cognitive score, indicating the more problems patients solved on the ToL the better they scored on the cognitive UHDRS. Total planning time was positively correlated with total number of solved problems and total number of breaks and negatively correlated with the UHDRS motor scale. All other associations were small and nonsignificant.

**Table 2:** Correlation matrix showing the significant associations of late-stage and early-stage patients’ characteristics with performance on the Tower of London

	Late-stage HD		Early-stage HD
	Total number of solved problems	Total planning time	Total number of solved problems
Disease duration	NS	NS	−.43
MMSE	NS	NS	.68
Premorbid IQ	NS	NS	.53
UHDRS motor scale	NS	−.42	−.59
UHDRS cognitive score	.60	NS	.65
Total number of solved problems	NS	.48	NS
Total number of breaks	NS	.52	NS

For all of correlations Pearson’s *r* was employed

For the group of controls, the total number of solved problems was significantly positively correlated with years of education and negatively correlated with age indicating that controls scored better with more years of education and with younger age. Total planning time was negatively correlated with age, and total number of breaks was negatively correlated with the number of solved problems. All other associations were nonsignificant.

## Discussion

In this study, we investigated differences in executive sub-domains between early- and late-stage HD patients compared to healthy controls.

In terms of accuracy of problem solving, controls showed better performance relative to early-stage HD patients, and early-stage HD patients showed better performance compared to late-stage HD patients. In contrast, for the planning time as well as the mean number of breaks taken during performance of the ToL, there were significant differences between controls and late- and early-stage HD patients. However, there were no significant differences between late- and early-stage HD patients. Furthermore, the total planning time of late-stage HD patients did not increase as the difficulty of the ToL conditions went up but remained constant throughout the test.

Accuracy, evaluated with total number of solved problems, is worse in late-stage HD patients compared to early-stage HD patients. Unschuld [19] and Watkins [18] both reported significantly impaired accuracy in their studies of ToL performance in early-stage HD patients compared to controls, which was also found in the current study. Significant differences of accuracy between early- and late-stage HD patients might be related to neuronal cell changes and cell death in the dorsolateral caudate head. In patients with HD, apoptosis in the caudate proceeds from dorsal to ventral and from medial to lateral with the earliest changes being seen in the medial paraventricular caudate, caudate tail, and dorsal putamen [25]. Indeed, those striatal structures are closely connected with the cortex via five parallel loops called the corticostriatal circuits [8]. It follows that the associative circuit, between the dorsolateral caudate (which degenerates in early stages of the disease) and the dorsolateral prefrontal cortex (DLPFC), is dysfunctional in the early course of the disease, while other circuits like the orbitofrontal circuit and the limbic circuit remain intact until more severe stages. In imaging studies of ToL performance in healthy controls, the DLPFC is recruited [26, 27]; accordingly early damage of the DLPFC may specifically lead to the progressive dysfunction of accuracy typically seen. Accuracy, being better in early-stage HD as in late-stage HD patients, does not seem to be influenced or compensated by other neuropsychological functions or brain changes. In early-stage HD patients, accuracy correlated with disease parameters like the MMSE scores and the scores on the UHDRS motor and cognitive scales. Subsequently, testing of accuracy during the ToL is a predictor for the specific cognitive function in early-stage HD patients. However, in late stages of HD the only significant relationship was found between accuracy and the cognitive scale score, all other correlations, which are seen in early-stage HD patients, were not detected. Therefore, in late-stage HD patients the number of solved problems on the ToL is no indicator for most of the clinical changes.

Patients’ and controls’ planning time was recorded from the moment of presentation of the start board and the goal board to the first movement; all participants had the instruction not to start until they had found the right solution for the problem. While healthy controls and early-stage HD patients subsequently required more time as task difficulty increased, the planning time (in other studies also called reaction time or latency) of patients in late stages of HD remained stable. This effect of the ToL is also seen in other patients with frontal pattern lesions [28–30] like in patients with Parkinson’s disease. The stable planning time in late-stage HD patients is explained by the development of impulsive behaviour during the course of the disease. Impulsivity and disinhibition are commonly found in HD patients [31–33], predominantly in later stages of the disease. This effect is also seen in other neuropsychological tests like the Stroop test: on this task HD patients regularly show impairment of impulsivity and disinhibition [29]. For the current study, it appears that patients in late stages are not able to control their impulsivity anymore, accordingly they start too early with the task before they have found the right solution. This is in agreement with the significantly higher number of breaks of late-stage HD patients compared to controls during six-move problems. Due to their impulsivity, late-stage patients start too early with the ToL and accordingly need more breaks to reflect the problem and solution once more. Furthermore, the current findings are supported by the positive correlation of total planning time with the total number of solved problems, indicating that late-stage patients who were able to suppress their impulsivity had a longer planning time and could consecutively solve more problems. Also in clinical practice, some late-stage HD patients manage to suppress their increasing impulsive behaviour and are able to carry out their activities of daily living without a major problem. Another behavioural problem commonly seen in HD patients in late stages is mental slowness. Even though we had expected to find influences of mental slowness in the assessment of planning time, this behavioural effect could not be displayed by our findings. In our study, the effects of impulsivity and disinhibition seem to outweigh those of mental slowness. As planning time reflects effects of impulsivity as well as mental slowness, both parameters cannot be disentangled. Regarding the assessment of planning time in early-stage HD patients, it appears that they are still able to control their impulsivity and manage to adhere to instructions given previously.

Early-stage HD patients only showed a significantly shorter planning time than controls for three-move problems. Also the ToL study of Unschuld and colleagues reported no significant difference between the planning times of early-stage affected patients, asymptomatic patients and controls [19]; additionally Watkins and colleagues did not find a significantly longer planning time for early-stage HD patients [18] when one-movement problems were excluded from the analysis. Summing up, the results of early- and late-stage HD patients vary in executive sub-domains.

### Limitations

In our study, all HD patients remained on their usual pharmacological treatment. As this study was mainly focusing on clinical features of HD, we chose to include patients on their usual treatment regimens to get a naturalistic depiction of reality.

None of the early-stage HD patients and two of late-stage HD patients took antipsychotics, and nine of early-stage HD patients and two of late-stage HD patients took antidepressants at the time of testing. Eighteen late-stage HD patients and one early-stage HD patient took both.

The findings of antipsychotics producing brain volume changes are controversial, and heir influence on brain volumes is still under debate. A review of Roiz-Stantianez [34], which included 41 MRI studies in schizophrenia, could not find a linear relationship between antipsychotic exposure and progressive brain changes. Up to now, it remains unclear whether basal ganglia volume changes in patients who are treated with antipsychotics are due to the underlying disease (for example schizophrenia) or due to antipsychotic treatment [34, 35]. For Huntington’s disease, to our knowledge, there are no studies which show acceleration of striatal volume decline under antipsychotic treatment. Moreover, we cannot rule out that antipsychotic treatment affected the executive sub-domains measured in our study as they are known to influence cognitive functions [36]. This has to be taken into careful consideration when interpreting our results.

Furthermore, our study results yield no information about the progression of executive dysfunctions in individuals, as we did not test the same patients during the course of the disease.

## Conclusion

Not all sub-domains of executive dysfunction in late-stage HD are reflected by early-stage HD pathology. Accordingly, studies conducted on cognitive function of presymptomatic patients and patients in early stages are not transferrable to patients in late stages. Furthermore, the current study is in line with former results, which suggested that the development of cognitive decline is not always comparable with that of topographical changes of the striatum. Our study has clinical implications because goal-directed behaviours in daily life rely on executive processes. Knowledge about the characteristics of executive dysfunction sub-domains in late- and early-stage HD will provide better advice to caregivers and patients.

Further studies on the development of frontal patterns in more advanced HD patients are needed to gather further insight into the complex system of changes of executive functions during the course of HD.

Our study has been approved by the ethics committee of the Medical University of Graz and has therefore been performed in accordance with the ethical standards laid down in the 1964 Declaration of Helsinki and its later amendments. All participants gave their informed consent prior to their inclusion in the study.
